# CD4^+^CD25^hi^FOXP3^+^ Cells in Cord Blood of Neonates Born from Filaria Infected Mother Are Negatively Associated with CD4^+^Tbet^+^ and CD4^+^RORγt^+^ T Cells

**DOI:** 10.1371/journal.pone.0114630

**Published:** 2014-12-22

**Authors:** Ulysse Ateba-Ngoa, Ghyslain Mombo-Ngoma, Eva Zettlmeissl, Luciën E. P. M. van der Vlugt, Sanne de Jong, Pierre-Blaise Matsiegui, Michael Ramharter, Peter G. Kremsner, Maria Yazdanbakhsh, Ayola Akim Adegnika

**Affiliations:** 1 Centre de Recherches Médicales de Lambaréné, BP 118, Lambaréné, Gabon; 2 Department of Parasitology, Leiden University Medical Center, Albinusdreef 2 2333 ZA Leiden, The Netherlands; 3 Institut für Tropenmedizin, Universität Tübingen, Wilhelmstraβe 27 D-72074 Tübingen, Germany; 4 Département de Parasitologie-Mycologie, Université des Sciences de la Santé, BP 4009, Libreville, Gabon; 5 Centre de Recherches Médicales de la Ngounié, Fougamou, Gabon; 6 Department of Medicine I, Division of Infectious Diseases and Tropical Medicine, Medical University of Vienna, Währinger Gürtel 18–20, 1090 Vienna, Austria; University of Cape Town, South Africa

## Abstract

**Background:**

Children who have been exposed *in utero* to maternal filarial infection are immunologically less responsive to filarial antigens, have less pathology, and are more susceptible to acquire infection than offspring of uninfected mothers. Moreover children from filaria infected mothers have been shown to be less responsive to vaccination as a consequence of an impairment of their immune response. However, it is not well known how *in utero* exposure to parasite antigens affects cellular immune responses.

**Methodology:**

Here, 30 pregnant women were examined for the presence of microfilaria of *Loa loa* and *Mansonella perstans* in peripheral blood. At delivery, cord blood mononuclear cells (CBMC) were obtained and the CD4^+^T cells were phenotyped by expression of the transcription factors Tbet, RORγt, and FOXP3.

**Results:**

No significant difference was observed between newborns from infected versus uninfected mothers in the frequencies of total CD4^+^T cells and CD4^+^T cells subsets including CD4^+^Tbet^+^, CD4^+^RORγt^+^ T and CD4^+^CD25^hi^FOXP3^+^ T cells. However, there was a negative association between CD4^+^CD25^hi^FOXP3^+^T cells and CD4^+^Tbet^+^ as well as CD4^+^RORγt^+^ T cells in the infected group only (B = −0.242, P = 0.002; B = −0.178, P = 0.013 respectively).

**Conclusion:**

Our results suggest that filarial infection during pregnancy leads to an expansion of functionally active regulatory T cells that keep TH1 and TH17 in check.

## Introduction

Parasitic infections are highly prevalent in the tropics and when present during pregnancy can affect the immune system of an unborn child directly, through transfer of parasites or antigens across the placenta [1]. The early priming of the fetal immune system by parasitic antigens, has been reported to lead to a relative impairment of the innate and adaptive immune response in the neonate and later in infancy [2]–[8]. As a consequence neonates born from parasite infected mothers are more susceptible to infection [3] and have a weaker response to vaccines administered during the first years of their life [9]. This is well illustrated in a report by Malhotra and colleagues who observed that children exposed to malaria in utero acquire a tolerant phenotype to Plasmodium falciparum blood stages antigens and have an increased susceptibility to malaria infection during childhood in comparison to their malaria unexposed relatives [3]. With respect to response to vaccines, a study comparing response to BCG vaccination between children from Malawi and the UK showed an inferior response to BCG in Malawian children suggesting that pre natal exposure to environmental factors such as microorganism and parasites might in part account for the difference in the Malawian and UK infants [9]. Among the infections that are highly prevalent in rural areas of the world are parasitic helminths, which are thought to exert strong immune modulatory effects [10].

In sub-Saharan Africa a high prevalence of filarial infections, such as Wuchereria bancrofti, Onchocerca Volvulus, Loa loa and Mansonella perstans is often observed in pregnant women [11]. Transplacental transfer of Wuchereria bancrofti [12], [13] or its antigens [1] from the mother to the fetus has been reported. In the case of filariasis, a number of studies have indicated that in utero exposure to maternal filarial infections can have consequences for the child after birth. Epidemiological studies have shown children from filaria infected mothers to be more susceptible to filarial infection [5], [14] and to have a higher risk of mother-to-child transmission of human immunodeficiency virus (HIV) [15] in comparison with children born to uninfected mothers. How filarial parasites alter the immune system of the fetus during pregnancy has not been studied extensively. Helminth infections in general, and filariasis in particular, are associated with the induction of a regulatory network that dampen strong immunological reactivities [10]. The role of this regulatory network has also been suggested during an in vitro study where the cellular responsiveness and the cytokine production of cord blood mononuclear cells (CBMCs) of newborns from filaria infected mothers were assessed [16]. These CBMCs were hyporesponsive to Onchocerca volvulus derived antigens, along with high production of the suppressive cytokine, IL-10 [16]. Studies on peripheral blood mononuclear cells (PBMCs) from adults have emphasized the association between filarial infection and regulatory T cells. For example Babu et al. reported that stimulation of PBMCs with live infective-stage larvae of Brugia malayi resulted in a more pronounced activation of the regulatory network in filaria infected subjects resulting in alterations in TH1 and TH2 responses [17]. Similarly, Wammes et al. observed lower responsiveness of T and B cells upon stimulation with B. malayi antigens, as well as lower secretion of TH1- and TH2-type cytokines in B. malayi infected patients presenting with lymphatic filariasis compared to their uninfected counterparts [18]. Interestingly the hypo-responsiveness was reversed following depletion of CD4+CD25hiFOXP3+ regulatory T cells suggesting their role in dampening T cell responses [18].

CD4+CD25hiFOXP3+ regulatory T cells (also known as natural T regulatory cells (nTregs)) together with adaptive T regulatory cells appear to be associated with human helminths infection [19]. The nTregs develop in the thymus at an early stage of the human fetal development from CD4+CD25hi thymocytes that can recognize self-antigens [20]. Adaptive regulatory T cells, which are thought to develop in the periphery in response to exogenous antigens, can also regulate effector T cells. FOXP3 has been described as the principal transcription factor of nTregs [21] required both for the development of nTreg and the maintenance of its suppressive function [22]. As for nTreg cells, the adaptive regulatory T cells can express FOXP3. Other T helper cells can also be characterized by transcription factors. This is the case for TH1- (expressing T-bet) and TH17- cells (expressing RORγt). A tight correlation between the level of transcriptional factors and cytokines secreted by terminally polarized T cells has been described, suggesting the use of transcription factors as a marker of Th cells polarization [23], [24]. However utilization of transcription factors in this sense is relatively recent and little data is available regarding their expression as well as their profiles in newborns from helminth-infected mothers.

We asked the question whether maternal filarial infection can alter the early balance between the CD4+T cell subsets that are known to be involved in immune responses to malaria parasites, namely the TH1 [25], [26] and TH17 [27]. Therefore, we analyzed in Gabon the expression of Tbet, RORγt and FOXP3 in CBMCs from neonates born to mothers infected with Loa loa and/or Mansonella perstans, comparing them to profiles seen in neonates from uninfected mothers.

## Materials and Methods

This study was carried out between May and August 2011 in Fougamou, a semi-rural town located in the center of Gabon, a sub-Saharan African country. This area is known to be endemic for blood-borne filaria (Loa loa and Mansonella perstans) as well as malaria [28]–[30]. Study participants were pregnant women. For our study, inclusion of participants was based on filaria infection. Therefore filaria infected women were asked to join the study, and for each infected woman an uninfected counterpart was included as well.

Infection status of the mother was determined during pregnancy for Schistosoma haematobium and microfilaria infection. On the other hand P. falciparum infection of the mother was assessed throughout the pregnancy, as well as at the time of delivery. Additionally the presence of Plasmodium falciparum was determined in the cord blood and the placenta. Filarial infection (Loa loa and Mansonella perstans) was diagnosed two months before the expected date of delivery by the Leucoconcentration method and parasite count was obtained by microscopy [31]. None of the mother was treated before delivery since the drugs to treat Loa loa and Mansonella perstans are not recommended during pregnancy. Plasmodium spp. infection status was determined based on a thick blood smears (TBS) made from 10 µl of blood and read by microscopy according to the Lambaréné method [32]. Diagnosis of S. haematobium infection was based on the detection of parasite eggs in the residue of 10 ml urine passed through a Millipore membrane filter, and examined by microscopy. Absence of infection was confirmed upon three negative results. Blood cell counts and hemoglobin level of the mother was obtained using the ABX Pentra 60 (HORBIA Medical).

**Figure 1:**
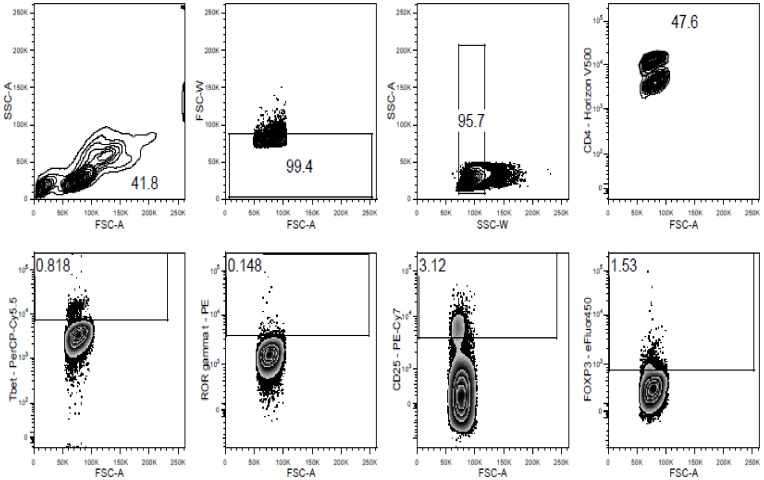
CBMC of neonates from filaria infected and uninfected mothers were isolated, fixed and stained with fluorescently labeled antibodies specific to surface markers (CD4, CD25) and intracellular transcription factors (FOXP3, Tbet, RORγT). Here we show an example of the gating strategy used for the identification of CD4^+^Tbet^+^, CD4^+^RORγT^+^ and CD4^+^CD25^hi^FOXP3^+^ T cells. Cells were first gated for lymphocytes (FSC-A vs SSC-A) and for singlets (FSC-A vs FSC-W and SSC-w vs SSC-A). Next, CD4+ cells were selected, to which Tbet, RORgt, CD25 and FOXP3 gating was applied. By combining these gates, Tbet+RORgt-FOXP3-, RORgt+Tbet-FOXP3- and CD25hiFOXP3+Tbet-RORgt- CD4+ T cells could be analysed.

Nine milliliters of venous cord blood was drawn after delivery in a heparinized tube. CBMCs were isolated within 24 hours using a Ficoll-Hypaque density gradient centrifugation as described elsewhere (10). CBMC were then fixed with the eBioscience transcription factor fixation and permeabilization kit (eBioscience, San Diego, CA, USA) as per manufacturer instructions. Fixed cells were stored in DMSO freezing medium at −80°C. Staining with fluorescently-labeled antibodies specific to T cell surface markers and to FOXP3, RORγt and Tbet transcription factors was performed for 30 minutes at 4°C. The antibodies used and their combinations are shown in Table 1, whereas the gating strategy is displayed in Fig. 1. Data were acquired using a BD FACSCanto II flow cytometer using BD FACSDiva software and analysed using FlowJo.

**Table 1:** Combinations of monoclonal antibodies used for the flow cytometry analysis.

Antibody	Specificity
CD4-V500*/Tbet-PerP5.5#	Tbet positive T cells
CD4-V500*/RoRgT-PE#	RORgT positive T cells
CD4-V500*/CD25-PE-Cy7*/FOXP3-Efluor450#	FOXP3 T-regulatory cells

*Beckton Dickinson Bioscience, San Jose, USA.

# eBioscience, Inc., San Diego, USA.

### Statistical analysis

IBM SPSS Statistics version 20.0 was used for statistical analysis. Graphs were created using the R graphing package “ggplot2” version 0.9.0. Differences in proportions between the two groups were assessed using the Chi squared test or the Fisher exact test when appropriate. Continuous data were compared with the Student t-test or the Mann-Whitney test when data were not normally distributed. The associations between CD4+CD25hiFOXP3+ T cells and CD4+Tbet+ T cells, CD4+RorγT+ T cells respectively were analyzed by linear regression analyses. The level of significance was set at 0.05.

### Ethics Statement

The study was approved by the “Comité d' Éthique Régional Indépendant de Lambaréné” (CERIL). The study protocol was explained to each mother and a signed inform consent was sought individually.

## Results

A total of 30 pregnant women and their newborns were included in this study. Inclusion was based on the filaria infectious status of the mother so as to have two equally sized groups of filaria infected and uninfected subjects. Both groups were comparable at the time of inclusion with respect to demographic characteristics as shown in Table 2. A total of 13 mothers experienced malaria during pregnancy [5 (53%) were in the group infected with filarial parasites and 8 (33%) in the filarial uninfected group, p = 0,269] but all of them were free of malaria at the time of delivery. Two mothers were found with S. haematobium (1 in each group).

**Table 2:** Baseline characteristic of the mother and the children enrolled in the study.

	Microfilaria negative	Microfilaria positive	p
Number of subjects	15	15	
**Characteristic of the mother**			
Age in years, median (IQR)	22 (19–28)	23 (21–32)	0.279#
*Multiparity status, n (%)*	15 (100%)	11 (73%)	0.099##
Hemoglobin level, mean (±SD)	11.08 (±1.48)	12.27 (±1.71)	0.052###
**Characteristic of the neonates**			
*Female, n (%)*	7 (47%)	2 (13%)	0.108##
Gestational age, median (IQR)	38 (36–40)	39 (37–41)	0.280#
Birth weight, median (IQR)	2850 (2470–3130)	2895 (2630–3140)	0.575#

# Mann-Withney test.

## Fisher exact test.

### Independent sample t-test.

Overall the mean percentage of CD4+T cells in CBMCs was comparable between newborn of filaria infected and uninfected mothers (respectively 47.7% vs 43.9% of CBMC, p = 0.344). Further characterization of T helper cells based on the signature of transcription factors showed no significant difference between the filaria infected and uninfected groups in the distribution of CD4+Tbet + (0.16% vs 0.10%, p = 0.086), CD4+RORγt+ (0.12% vs 0.14%, p = 0.693) or CD4+CD25hiFOXP3+T cells (2.5% of CBMC vs 2.04% respectively, p = 0.210).

**Figure 2:**
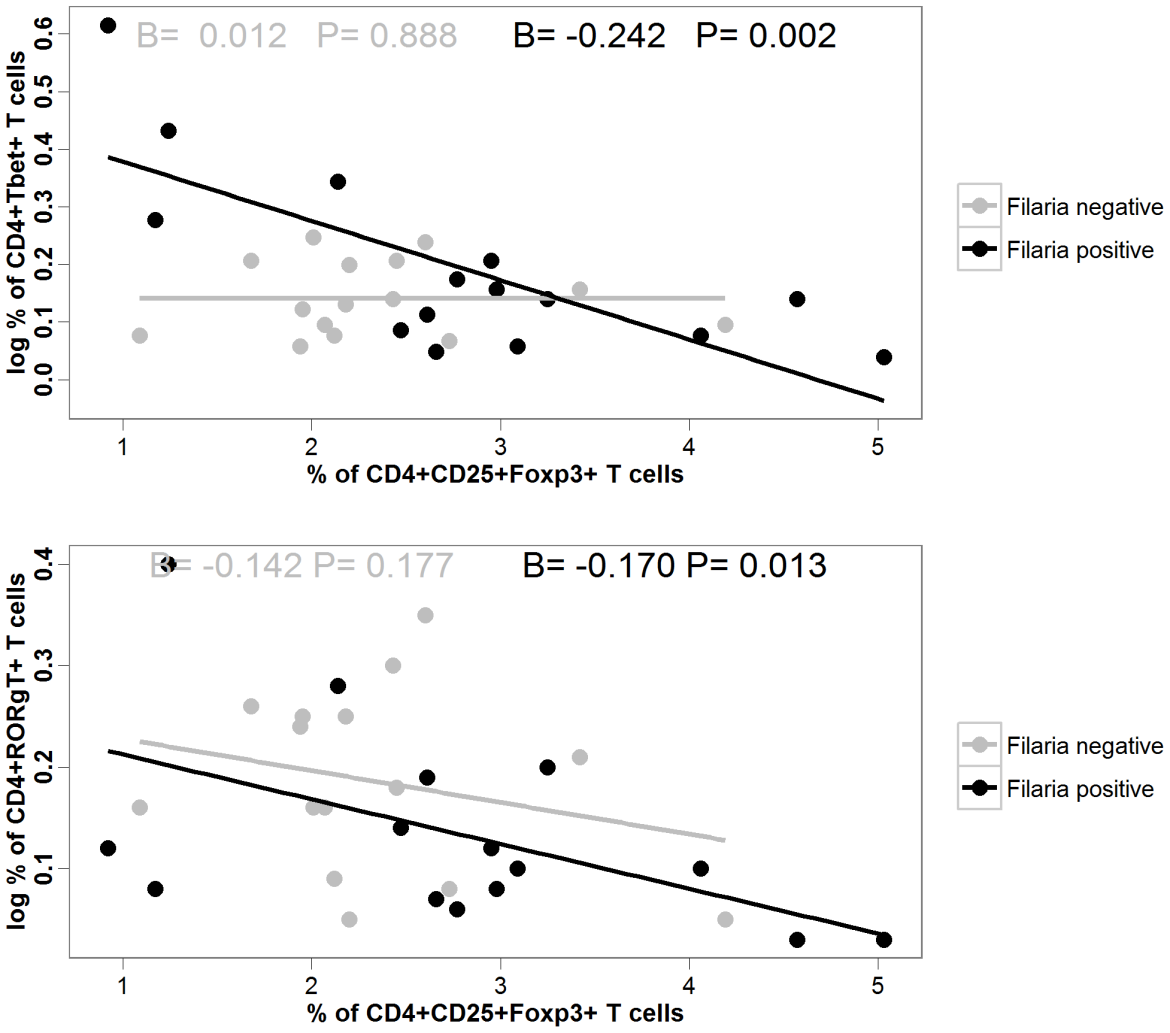
The relation between CD4^+^CD25^hi^FOXP3^+^ T cells and CD4^+^Tbet^+^ (upper panel) as well as CD4^+^CD25^hi^FOXP3^+^ T cells and CD4^+^RORγT^+^ T cells (lower panel) of CBMC of neonates from filaria negative (in grey) and filaria positive (in black) mothers assessed by a linear regression analysis. Each dot shows value from a single subject while the solid lines represent the regression lines of the model. The strength of the association between two variables is given by the value of the regression coefficient beta (β) value in each graph. A positive β value indicates a positive association between the variables in the model while a negative β value indicates a negative association. P values are given to indicate the statistical significance of the associations.

In order to assess the association between regulatory T cells and the different T helper subsets, we examined the association between CD4+CD25hiFOXP3+ T cells and CD4+ Tbet+ T cells, CD4+RORγt+ T cells respectively through a linear regression analysis. Analyzing all subjects together, we observed a negative association between Treg and CD4+Tbet+T cells (B = −0.149, 95% CI = −0.256 to −0.043, p = 0.008) or CD4+RORγT+T cells (B = −0.175, 95% CI = −0.275 to −0.074, p = 0.001). Interestingly when stratifying our study subjects by their infectious status we observed that the negative association between CD4+CD25hiFOXP3+ Treg cells and the population of TH1 and TH17 cells was only significant in the offspring of microfilaria infected mothers (Fig. 2).

## Discussion

This exploratory study was designed to assess the effect of maternal filarial infection on the neonatal T helper cells that are known to be involved in malaria driven immune responses, TH1 and TH17, using transcription factors that are now used as hallmarks of T helper cells polarization. To this end we measured the percentage of CD4+ T cells and CD4+ T cells expressing Tbet, RORγt and FOXP3 in CBMCs collected from offspring of filaria infected and uninfected mothers. We did not find a significant effect of maternal filarial infection on the percentage of Tbet+, RORγt+, CD25hiFOXP3+ CD4+T cells, nor on the level of expression of these transcription factors (data not shown).

Treg cells are important for maintaining immune homeostasis, especially during the course of an infection. Consequently, an expanded Treg compartment has been described in subjects that are chronically infected by parasites such as filarial worms [33]. Although, we observed a trend toward an increase of the percentage of Treg cells in CBMC of neonates from filaria infected mothers, this was not statistically significant. When we analyzed how Treg cells were associated with other cell subsets, we observed that they were negatively correlated with Tbet+ and RORγt+ CD4 T cells. Importantly, this was only seen in the filaria infected group and not in the cells of CBMC of neonates born to uninfected mothers suggesting a stronger functional activity of these cells in infected subjects. In line with our finding, a study reported by Wammes et al., compared both frequency and function of CD4+CD25hiFOXP3+ regulatory T cells in geohelminths infected and uninfected individuals [34]. This study found that although the frequency of regulatory T cells was similar between the two groups, their suppressive activity was more pronounced in geohelminths infected subjects [34]. Together with our data this result may suggest that activation of CD4+CD25hiFOXP3+ regulatory T cells occur upon exposure of the cells to parasite antigens endowing them with strong functional capacity.

The obvious limitation of our study is the small sample size, which may have prevented the detection of significant differences in percentages of TH1, TH17 and Treg cells in children born to infected and uninfected mothers. Despite this, we could by using a regression model show that in offspring from filaria infected mothers Treg cells could alter effector T cell expansion as described in adults [34]–[36]. When studying regulatory T cells in circumstances where it is not possible to assess their functional capacity (for example in resource poor settings), it might be useful to analyze relationships between their number and outcomes such as other cell subsets that these cells could control or cytokines produced by effector cells.

Altogether our finding that in children born to mothers infected with filarial worms have regulatory T cells that are negatively associated with TH1 or TH17 cells, may have practical implications, as an alteration of effector T cell responsiveness in neonates from helminth infected mothers may lead to a poor immunologic response to vaccines that are usually administered during their first years of life.

## Supporting Information

S1 Dataset **Filiaria Neonate.**
(TXT)
