# Correction: CD4^+^CD25^hi^FOXP3^+^ Cells in Cord Blood of Neonates Born from Filaria Infected Mother Are Negatively Associated with CD4^+^Tbet^+^ and CD4^+^RORγt^+^ T Cells

**DOI:** 10.1371/journal.pone.0119397

**Published:** 2015-03-26

**Authors:** The PLOS ONE Staff

The fifth author’s name is spelled incorrectly. The correct name is: Sanne E. de Jong. The correct citation is: Ateba-Ngoa U, Mombo-Ngoma G, Zettlmeissl E, van der Vlugt LEPM, de Jong SE, Matsiegui PB, et al. (2014) CD4+CD25^hi^FOXP3+ Cells in Cord Blood of Neonates Born from Filaria Infected Mother Are Negatively Associated with CD4+Tbet+ and CD4+RORγt+ T Cells. PLoS ONE 9(12): e114630. doi:10.1371/journal.pone.0114630
